# Correlation Analysis Between Magnetic Resonance Imaging-Based Anatomical Assessment and Behavioral Outcome in a Rat Contusion Model of Chronic Thoracic Spinal Cord Injury

**DOI:** 10.3389/fnins.2022.838786

**Published:** 2022-04-21

**Authors:** Cong Xing, Zeyu Jia, Haodong Qu, Song Liu, Wang Jiang, Hao Zhong, Mi Zhou, Shibo Zhu, Guangzhi Ning, Shiqing Feng

**Affiliations:** ^1^Department of Orthopedics, Tianjin Medical University General Hospital, Tianjin, China; ^2^International Science and Technology Cooperation Base of Spinal Cord Injury, Tianjin, China; ^3^Tianjin Key Laboratory of Spine and Spinal Cord Injury, Tianjin, China; ^4^Department of Radiology, Tianjin Medical University General Hospital, Tianjin, China

**Keywords:** MRI, sagittal T2WI, behavioral testing, correlation analysis, spinal cord injury, rat thoracic spine

## Abstract

Although plenty of evidences from preclinical studies have led to potential treatments for patients with spinal cord injury (SCI), the failure to translate promising preclinical findings into clinical advances has long puzzled researchers. Thus, a more reliable combination of anatomical assessment and behavioral testing is urgently needed to improve the translational worth of preclinical studies. To address this issue, the present study was designed to relate magnetic resonance imaging (MRI)-based anatomical assessment to behavioral outcome in a rat contusion model. Rats underwent contusion with three different heights to simulate various severities of SCI, and their locomotive functions were evaluated by the grid-walking test, Louisville swim scale (LSS), especially catwalk gait analysis system and basic testing, and Basso, Beattie, Bresnahan (BBB) score. The results showed that the lesion area (LA) is a better indicator for damage assessment compared with other parameters in sagittal T2-weighted MRI (T2WI). Although two samples are marked as outliers by the box plot analysis, LA correlated closely with all of the behavioral testing without ceiling effect and floor effect. Moreover, with a moderate severity of SCI in a contusion height of 25 mm, the smaller the LA of the spinal cord measured on sagittal T2WI the better the functional performance, the smaller the cavity region and glial scar, the more spared the myelin, the higher the volatility, and the thicker the bladder wall. We found that LA significantly related with behavior outcomes, which indicated that LA could be a proxy of damage assessment. The combination of sagittal T2WI and four types of behavioral testing can be used as a reliable scheme to evaluate the prognosis for preclinical studies of SCI.

## Introduction

Spinal cord injury (SCI) is a refractory disease of the central nervous system that not only causes permanent disabilities for individuals but also represents a huge burden to families and healthcare systems (Ahuja et al., 2017; Huang et al., 2020). The most common causes of SCI are traffic accidents and falls; degenerative cervical myelopathy can also progress to incomplete SCI if not recognized and treated timely (Zipser et al., 2021). According to a global investigation of SCI, falls and road injuries are the most common causes in most countries and regions (James et al., 2019).

SCI usually leads to irreversible neurodegenerative changes such as demyelination and axon damage (Seif et al., 2020). It is generally recognized that underlying pathophysiological processes of SCI can be divided into two stages, primary and secondary damage (Fan et al., 2018). Overwhelming cell death and degeneration promote the cystic cavity formation, which contains fibrous connective tissue, plenty of extracellular fluid, and activated microglia/macrophage (Norenberg et al., 2004). The cystic cavities aggregate to become an impenetrable barrier that block directed axonal regrowth and cell migration (Rowland et al., 2008). Reactive astrocytes proliferate and closely arrange in the perilesional zone around the cystic cavities, called glial scar, which also potently restricts axon regeneration and plasticity (Barnabé-Heider et al., 2010; Göritz et al., 2011). Therefore, cystic cavities along with glial scar act as the anatomical barrier to the regeneration following SCI.

Besides diagnosis and classification of SCI, neurological examinations and spinal imaging are also necessary in evaluating the degree of recovery. Magnetic resonance imaging (MRI) is the most common radiological tool for investigating damage to the SCI (Le et al., 2015; Aarabi et al., 2017; Dalkilic et al., 2018). MRI is sensitive to the intramedullary lesion, and the change of sagittal T2-weighted MRI (T2WI) can reflect various processes, including cytotoxic edema, hemorrhage, inflammation, and cyst cavitation dependent on the timing of assessments. Therefore, MRI has been utilized to diagnose acute SCI and predict functional recovery in the chronic phase (Aarabi et al., 2017). Crucially, lesion size and signal intensity might be sensitive to the change of sensorimotor function; these quantitative neuroimaging parameters can serve as both diagnostic and prognostic tools at all stages of SCI. Thus, parameters of sagittal T2WI could serve as important markers for clinical trials and preclinical trials to evaluate the recovery after injury (Huber et al., 2017; Cadotte et al., 2018). In recent animal studies, T2WI has been used to observe the extent of the lesion for evaluating the recovery after SCI (Peng et al., 2019; Robac et al., 2021).

The generally accepted examination of sensorimotor function is according to the Neurological Classification of Spinal Cord Injury (ISNCSCI) that has been proven to be reliable for the evaluation of injury severity (Kirshblum et al., 2011, 2020). Similar to a clinical study, evaluation of behavioral outcome is also crucial in preclinical animal experiments for developing therapy strategies. In addition to the methods that rely entirely on experienced researchers, like Basso, Beattie, Bresnahan (BBB) open-field locomotor scale, grid-walking test, and Louisville swim scale (LSS), the catwalk gait analysis system is also widely used as an objective behavioral testing to evaluate recovery of locomotive function in rat models (Behrmann et al., 1992; Basso et al., 1995; Schucht et al., 2002; Hamers et al., 2006; Smith et al., 2006a; Walker et al., 2019; Fouad et al., 2020).

Although lesion size has been recognized as the most accurate marker of the SCI, some researches have shown that similar lesion sizes may produce different grades of functional impairment or recovery in both clinical trials and preclinical trials; when the magnitude of lesion pathology does not the match function status, neurobiologists called this phenomenon the “neuroanatomical–functional paradox.” (Marino et al., 1999; Schucht et al., 2002; Fawcett et al., 2007; Hurd et al., 2013). This neuroanatomical–functional paradox will also preclude the translation from basic research to clinical treatment (Fouad et al., 2021). Thus, the primary objectives of this study were to (a) explore the association between the parameters measured on sagittal T2WI and behavioral outcome evaluated by several experience-dependent and objective methods and (b) demonstrate whether a similar neuroanatomical–functional paradox exists in the rat contusion model of SCI.

## Materials and Methods

### Animals and Experimental Groups

A total of 22 adult female Wistar rats weighing 200 ± 10 g (Charles River Laboratories, Beijing, China) were included in this study. The rats were maintained in a standard condition (temperature of 22 ± 1°C, air humidity of around 55%) with a 12:12 h light–dark cycle and freely accessed food and water. All experiments of this study were authorized by the Ethics Committee of the Institute of Radiation Medicine, Chinese Academy of Medical Sciences & Peking Union Medical College (approval number: IRM-DWLL-2021111) and conform to all guidelines regarding the use of animals of the National Institutes of Health in research.

At first, a subset of rats (*n* = 15) was randomly divided into three groups equally, SCI-mild (SCI-mil) group, SCI-moderate (SCI-mod) group, and SCI-severe (SCI-sev) group.

In translational SCI studies, the animals of each group generally underwent the same severity of injury before being given different interventions, and the moderate injury was used more frequently. In order to simulate the grouping comparison of translational studies, extra rats (*n* = 7) were required in the second stage, which underwent the same injury with subjects of the SCI-moderate group. A total of 12 rats were dichotomized into SCI-moderate-smaller group (SCI-mod-s) and SCI-moderate-larger group (SCI-mod-l) by the median of the lesion area measured on sagittal T2WI.

### Experimental Design

**FIGURE 1:**
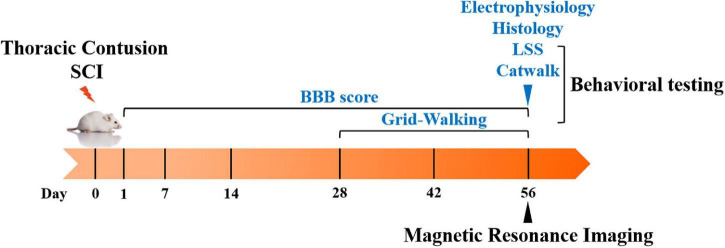
Schematic diagram of the experimental design.

The timeline of the experiment is illustrated in Figure 1. The rats underwent contusion injury of the T10 segment with three different severities depending on the groups. The BBB open-field locomotor scale was administered at the first day post-injury and weekly thereafter until 56 days post-injury, and the grid-walking test was performed at 28, 42, and 56 days post-injury. LSS and the catwalk gait analysis system were conducted at 56 days post-injury. MR images were also acquired at 56 days post-injury; the lesions were segmented and calculated from the T2WI datasets. The correlation analysis between the lesions and behavior was performed by Spearman’s rank correlation coefficient test. The methods of histology and electrophysiology were also used to observe the subtle difference between SCI-mod-l group and SCI-mod-s group at the end of the experiment.

### Spinal Cord Injury

The thoracic contusion SCI model was established using the modified Allen method by the MASCIS Impactor Model III (W. M. Keck Center, Rutgers University, United States). Each animal was initially weighed and anesthetized with isoflurane inhalation anesthesia (2.5% for induction and 2.0% for maintenance). Along the dorsal skin, an 8-mm midline incision was made after local shaving and sterilization, paraspinal muscles were separated to expose the T10 laminae. Contusions of the spinal cord were conducted by dropping the impactor from the heights of 12.5 mm (mild), 25.0 mm (moderate), and 50.0 mm (severe) after the laminectomy of T10 was performed. Postoperatively, animals received cefotaxime (10 mg/kg, i.h.) and 0.9% saline (5 ml/kg, i.h.) for 3 days to prevent infections and dehydration. Bladders were expressed manually at least twice per day until spontaneous voiding was regained.

### Magnetic Resonance Imaging

**FIGURE 2:**
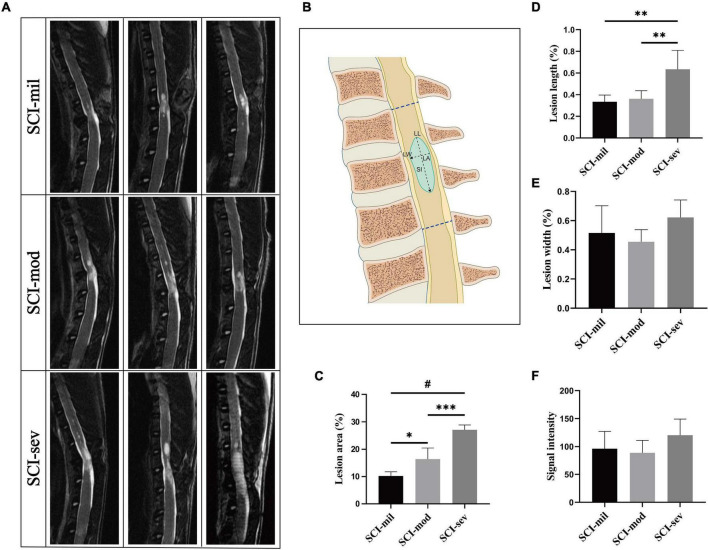
MRI acquisition and analysis of lesion region. **(A)** Sagittal T2-weighted images of rats in three groups. **(B)** Schematic drawing of the parameters, which include lesion area (LA), lesion length (LL), lesion width (LW), and signal intensity (SI). LA was calculated as hyperintense region/overall region of T9–T11 vertebral canal × 100%. **(C–F)** Comparison among three groups on LA, LL, LW, and SI, respectively. *n* = 5 per group (*p < 0.05; ^**^p < 0.01; ^***^p < 0.001; ^#^*p* < 0.0001).

MR images of the spinal cord were acquired on a 3.0 T spectrometer (Discovery MR750, GE Healthcare, United States). Animals were anesthetized and placed in the supine position before MRI scans at 8 weeks post-injury. Fast relaxation fast spin echo (FRFSE) pulse sequences were used for the acquisition of T2-weighted images. Specifically, the FRFSE T2-weighted sequence had the following acquisition parameters: TR/TE = 3,000/110 ms, image matrix = 320 × 224, FOV = 6 mm, slice thickness = 2 mm, spacing = 0.5 mm, echo train length = 21, and NEX = 4. The lesions were segmented by the thresholding method of gray value (≥ 85) from sagittal T2-weighted images and calculated by ImageJ software (version 1.8.0, National Institutes of Health, United States). The measurement of lesion indicators is shown in Figure 2B, where the lesion area (LA) represents the proportion of the lesion area in the T9–T11 spinal cord; lesion length (LL) and lesion width (LW) represent the maximum longitudinal and transverse diameters of the lesion area, respectively; and signal intensity (SI) represents the signal strength of the lesion area.

### Behavioral Testing

To evaluate impairment and functional recovery of animals after SCI, several locomotor behavioral assessment methods were applied in this research. All the tests were performed at the same time of the day and were graded by two blinded observers with professional training.

#### Basso, Beattie, Bresnahan Locomotor Score

The BBB test was performed at the first day after injury and weekly thereafter until the animals were sacrificed. During the test, rats were placed into an open field and walked freely during a 5-min observation period; hindlimb movement was evaluated using a rating scale ranging from 0 to 21 points. The final score was the average of the scores obtained from the two observers.

#### Grid-Walking Test

To further explore the recovery of fine motor coordination, the grid-walking test was performed biweekly from 4 to 8 weeks post-injury. Animals were placed and freely walked for at least 5 min on a mental elevated device with a grid size measuring 3 cm by 3 cm; images were recorded by using a camera placed underneath the grid. Each hindlimb of the rats that fell into the grid holes were counted as a stepping error; the proportion of stepping errors was calculated as stepping errors/total steps × 100%.

#### Louisville Swim Scale

A 60-cm-long, 33-cm-wide, and 38-cm-deep clear chamber, with a ramp consisting of an acrylic plate and a chloroprene rubber that is 5 mm thick on one side, is used as swimming pool. The pool was filled to a depth of 30 cm with warm tap water (28–30°C), and each rat was trained twice to swim from one side to the other side and reach the ramp to exit the pool before the final test at 8 weeks post-injury. Forelimb dependency, hindlimb movement and alternation, trunk stability, and body angle were analyzed and evaluated on the basis of the LSS scoring sheet ranging from 0 to 17 points; each rat was tested twice to get the average score.

#### Catwalk Gait Analysis System

The catwalk gait analysis system (Noldus Information Technology, the Netherlands) was used as an objective method to assess the locomotor gait dynamics of the rats in this research. For the CatWalk XT (version 10.6) analyses, the animals were trained on the device for at least two times before surgery. On the premise of the same calibration parameters in each group, the locomotor activities of rats were recorded at least three times. Several indicators were assessed in the test, including swing (the duration in seconds of no contact of a paw with the glass plate), max contact area (the surface area of the print at max contact expressed), regularity index (the number of normal step sequence patterns relative to the total number of paw placements), and print position (the mean values for the right paws and the left paws).

### Electrophysiological Detection

The rats were anesthetized, and the skin was prepared for insertion of the probe electrode before the motor evoked potentials (MEPs) were recorded using an electrophysiological device at 8 weeks post-injury. For the measurement of the MEP, each stimulating electrode was inserted under the skin above the nose, the recording electrodes were placed in the same bundle of muscle of calf, and the electrode to the ground was linked with the dorsal skin. The MEPs of each rat were recorded at least five times.

### Histology

#### Hematoxylin-Eosin Staining

Hematoxylin-Eosin (HE) staining was used to assess the contusion area and the function of urinary bladder in two SCI-moderate groups after the animals were sacrificed at 8 weeks post-injury. Both spinal cord samples and bladder samples were embedded in paraffin and sliced into sections. Then the sections were stained with an HE staining kit (Solarbio, Beijing, China) and were observed under an optical microscope (IX73, Olympus, Tokyo, Japan). The cavity area of the damaged region and bladder wall thickness were measured by ImageJ software for comparison between the two groups.

#### Immunofluorescent Staining

For IF staining, anesthetized rats were transracially perfused with pre-cooled 0.9% saline and 4% paraformaldehyde in turn. The 6-μm-thick longitudinal frozen sections of the spinal cord samples were manufactured to be probed with anti-glial fibrillary acidic protein (GFAP) antibody (1:500, Bs-0199r, Bioss, Beijing, China) and goat anti-rabbit IgG Alexa 555 (1:500, ab150086, Abcam, Cambridge, United Kingdom) for observing the area of the scar tissue, and the staining results were observed by a Leica fluorescence microscope (DMi8, Leica, Germany).

#### Myelin Staining

For assessing the integrity of the myelin, the cross sections were brought to room temperature, then rehydrated in PBST (PBS solution with 0.2% Triton X-100) for at least 20 min, and stained with FluoroMyelin™ Green stain solution (1:300, F34651, Invitrogen, OR, United States) for 20 min. When staining was complete, sections have been washed three times for 10 min each with PBS solution. Finally, images of the myelin staining were captured under a fluorescence microscope (DMi8, Leica, Germany).

### Statistical Analysis

The data were analyzed by using GraphPad Prism, version 8.0.2, software (San Diego, CA, United States) and SPSS, version 23.0, statistical software package (SPSS Inc., Chicago, IL, United States). Groups were compared using one-way analysis of variance (ANOVA) with Tukey’s honestly significant difference (HSD) *post-hoc* test. Statistical difference among the SCI-mod-s group and SCI-mod-l group was evaluated by unpaired Student’s *t*-test. The correlation between the LA of the spinal cord and behavioral data was analyzed using Spearman’s rank correlation coefficient test, and possible outlying observations were detected by box plot analysis and marked in the graphs. Outliers were determined by interquartile range (IQR), more than upper quartile + 1.5 IQR or less than lower quartile - 1.5 IQR would be identified as outliers. All data were presented as the means ± SEM, and the *P*-values < 0.05 were considered to indicate a statistically significant difference, except for correlation analysis. In correlation analysis, multiple comparison correction with Bonferroni control was performed, and the threshold was set as 0.007 (0.05/7).

## Results

Of the animals with a highly accurate and controlled impacts with three heights, all showed no observable hindlimb movement (BBB score of 0) at the day post-injury; spontaneous voiding was regained before 7 days post-injury; and there were no other complications. To investigate the correlation between the anatomical evaluation and functional recovery, MRI and behavioral testing were performed (Supplementary Table 1).

### Magnetic Resonance Imaging Morphometry of the Spinal Cord in Sagittal T2-Weighted Images

Different lesion regions of the spinal cord in three groups were observed in sagittal T2-weighted images (Figure 2A). As shown in Figure 2B, LA, LL, LW, and SI of the spinal cord in each rat were measured and calculated to assess the injury (Figure 2B). As clearly illustrated by the bar graph, a significant growing trend of LA from the SCI-mil group and SCI-mod group to the SCI-sev group has been observed (Figure 2C). Although a similar trend was observed in the results of LL, there was no statistical difference between the SCI-mil group and SCI-mod group (Figure 2D). Besides that, there was little or no difference in the LW and SI among the groups (Figures 2E,F). The data suggested that the lesion size increases with the increase of the height of contusion, and the value of LA may be the best indicator for damage assessment after SCI.

### Correlation Between Magnetic Resonance Imaging and Experience-Dependent Behavioral Testing

**FIGURE 3:**
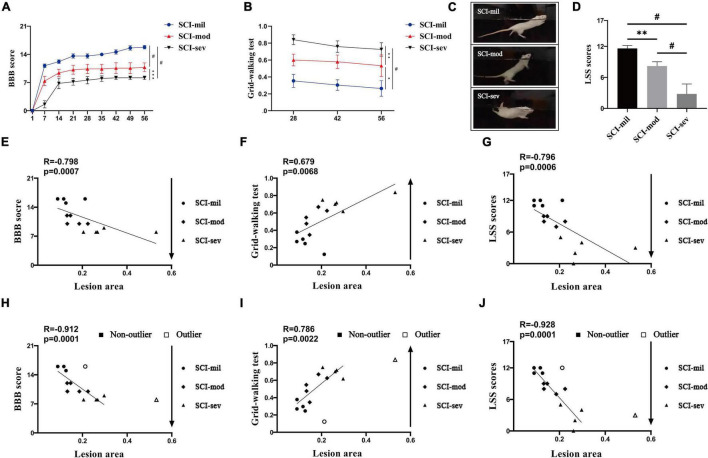
Experience-dependent methods of behavioral testing and Spearman’s rank correlation coefficient test. Various levels of spontaneous recovery, comparison among three groups on **(A)** BBB score and **(B)** grid-walking test, were evaluated by experience-dependent methods in rats that underwent contusion of three different severities. **(C,D)** Photographs of swimming test and comparison among three groups on LSS scores. *n* = 5 per group (*p < 0.05; ^**^p < 0.01; ^***^p < 0.001; ^#^*p* < 0.0001; ns represents no statistical significance). Spearman’s rank correlation coefficient test. **(E–G)** Correlation analysis between lesion area and experience-dependent methods of behavioral testing including outliers. **(H–J)** Correlation analysis between lesion area and experience-dependent methods of behavioral testing after removing outliers.

The three groups of animals with the contusion of different heights had shown various levels of spontaneous recovery in the experience-dependent behavioral testing. As shown in Figures 3A,B, the average recovery in the BBB open-field locomotor score during a time frame of 8 weeks and the ability to cross the grid walk during a time frame of 4 weeks reveals significant differences among the three groups. Similarly, swimming performance of the animals had gotten worse as the contusion height increased (Figures 3C,D). The BBB score, the grid-walking test, and the LSS at 56 days post-injury all showed a strong correlation with LA of the spinal cord observed in sagittal T2-weighted images (*R* = -0.798, 0.679, and -0.796, respectively; *P* < 0.001, *P* < 0.01, *P* < 0.001) (Figures 3E–G). However, two samples are marked as outliers by the box plot analysis of LA although their behaviors were similar to others in the same group. The results of the analysis had also shown a strong correlation after removal of outliers (*R* = -0.912, 0.786, and -0.928, respectively; *P* < 0.0001, *P* < 0.01, *P* < 0.0001) (Figures 3H–J).

### Correlation Between Magnetic Resonance Imaging and Catwalk Gait Analysis System

**FIGURE 4:**
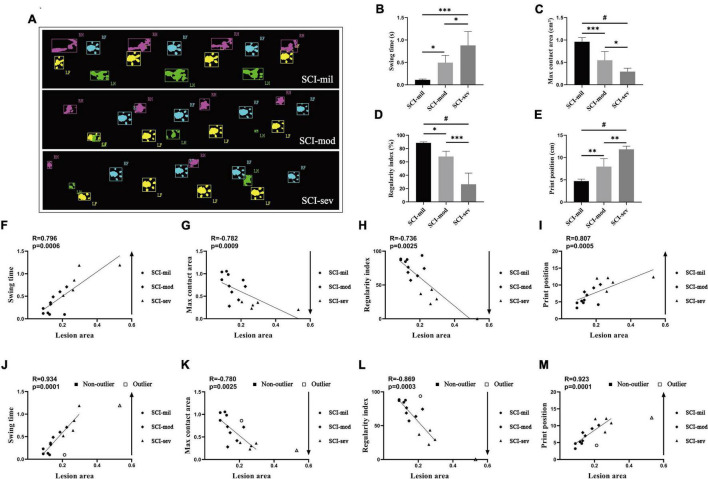
Catwalk test and Spearman’s rank correlation coefficient test. **(A)** Photographs of catwalk test in three groups. Various levels of spontaneous recovery, comparison among three groups on **(B)** swing time, **(C)** max contact area, **(D)** regularity index, and **(E)** print position, were evaluated by the catwalk test in rats that underwent contusion of three different severities. *n* = 5 per group (*p < 0.05; ^**^p < 0.01; ^***^p < 0.001; ^#^*p* < 0.0001; ns represents no statistical significance). **(F–I)** Correlation analysis between lesion area and indicators of the catwalk including outliers. **(H–J)** Correlation analysis between lesion area and indicators of catwalk after removing outliers.

The acquisition of many important data of catwalk gait analysis system requires rats to walk with weight supported by plantar (a BBB score of 11). According to the recovery of rats, the catwalk gait analysis system was used as an objective behavioral testing to evaluate the locomotive function only at 8 weeks post-injury. Similar to the results of experience-dependent behavioral testing, the data of the catwalk gait analysis system had also shown various levels of spontaneous recovery (Figure 4A). All of swing, max contact area, regularity index, and print position reveal significant differences among the three groups (Figures 4B–E) and showed a strong correlation with LA of the spinal cord observed in sagittal T2-weighted images (*R* = 0.796, -0.782, -0.736, and 0.807, respectively; *P* < 0.001, *P* < 0.001, *P* < 0.001, *P* < 0.0001) (Figures 4F–I). Without the max contact area, the results of the analysis also had shown a stronger correlation after removal of outliers (*R* = 0.934, -0.869, and 0.923, respectively; *P* < 0.0001, *P* < 0.001, *P* < 0.0001) (Figures 4J–M).

Reflecting our data, these results point to a strong correlation between all of behavioral testing and LA of the spinal cord observed in sagittal T2-weighted images (Table 1), even though a similar magnitude of lesion pathology does not the match the function status also existing in the rat model.

**TABLE 1:** Relationships between lesion area and behavior testing.

Lesion area vs.	Correlation coefficient, *R* (*p*-value)	
	Include outliers	Remove outliers
BBB score	-0.7984 (0.0007)	-0.9120 (0.0001)
Grid-walking test	0.6786 (0.0068)	0.7857 (0.0022)
LSS scores	-0.7961 (0.0006)	-0.9282 (0.0001)
Swing time	0.7964 (0.0006)	0.9341 (0.0001)
Max contact area	-0.7821 (0.0009)	-0.7802 (0.0025)
Regularity index	-0.7361 (0.0025)	-0.8691 (0.0003)
Print position	0.8071 (0.0005)	0.9231 (0.0001)

### Comparison Between SCI-Mod-s Group and SCI-Mod-l Group on Behavioral Testing

Seven rats were added to the SCI-moderate group in the following experiments.

**FIGURE 5:**
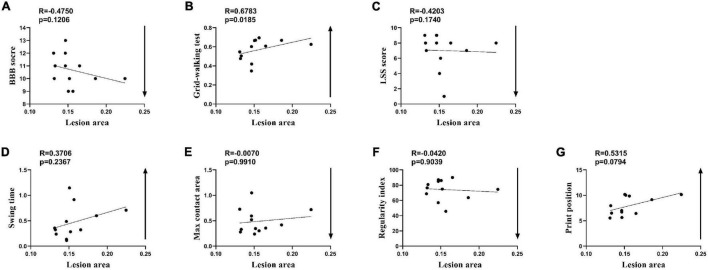
Spearman’s rank correlation coefficient test of the SCI-moderate group after seven additional rats were added. **(A–G)** Correlation analysis between lesion area and indicators of behavior (*n* = 12.)

**FIGURE 6:**
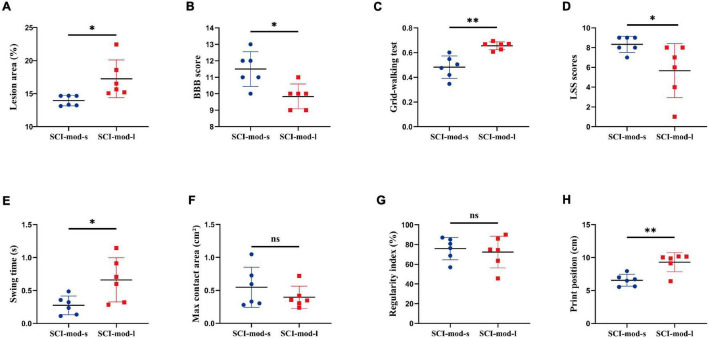
Comparison between the SCI-mod-s group and SCI-mod-l group on **(A)** lesion area, **(B)** BBB score, **(C)** grid-walking test, **(D)** LSS scores, **(E)** swing time, **(F)** max contact area, **(G)** regularity index, **(H)** print position. *n* = 6 per group (*p < 0.05; ^**^p < 0.01; ns represents no statistical significance).

Because of the narrow range of LA and value of behavioral recovery, *R* was not significant in the correlation analysis and only some correlated trend was observed (Figure 5). Permutation testing verified that LA was a reliable observation for grouping the rats of the SCI-mod group. Therefore, a total of 12 rats were dichotomized into two subgroups (SCI-mod-s group and SCI-mod-l group) by median of LA (Supplementary Table 2). The SCI-mod-s group had lower LA of spinal cord than the SCI-mod-l group (0.14 ± 0.01% and 0.17 ± 0.03%, respectively; *P* < 0.05) (Figure 6A).

Consistent with the hypothesis, the SCI-mod-s group had a lower proportion of stepping errors (0.48 ± 0.08% and 0.66 ± 0.03%, respectively; *P* < 0.01), higher BBB score (11.50 ± 0.96 and 9.83 ± 0.69%, respectively; *P* < 0.05), and higher LSS scores (8.33 ± 0.75 and 5.67 ± 2.49, respectively; *P* < 0.01) than the SCI-mod-l group (Figures 6B–D). Similar to experience-dependent behavioral testing, swing time (0.28 ± 0.13 and 0.66 ± 0.31 s, respectively; *P* < 0.05) and print position (6.56 ± 0.82 and 9.30 ± 1.33 cm, respectively; *P* < 0.01) of the catwalk gait analysis system were also lower in the SCI-mod-s group (Figures 6E,F), but there was no statistical difference in maximum contact area and regularity index between two groups (Figures 6G,H). It seems that the smaller the LA of the spinal cord, the better the functional performance, but maximum contact area and regularity index may not be sensitive indicators to evaluate subtle differences.

### Comparison Between the SCI-Mod-s Group and SCI-Mod-l Group on Histology and Electrophysiology Detection

**FIGURE 7:**
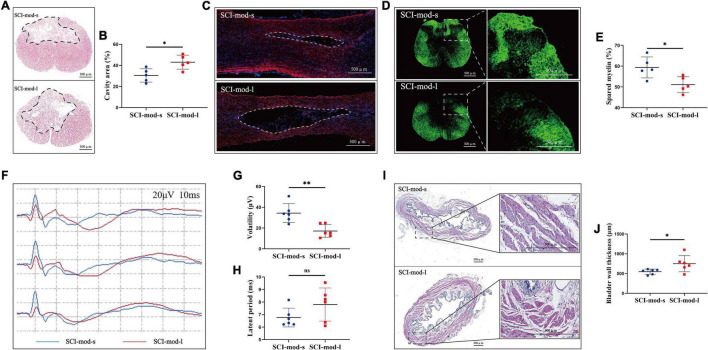
Comparison between the SCI-mod-s group and SCI-mod-l group on **(A,B)** cavity region; **(C)** glia scar; **(D,E)** spared myelin; **(F–H)** electrophysiological detection; **(I,J)** thickness of bladder wall (*p < 0.05; ^**^p < 0.01; ns represents no statistical significance).

To confirm the concordance between MRI-based anatomical assessment and histological analysis, we performed HE staining, myelin staining, and IF staining on the spinal cord sections. Consistent with LA of the spinal cord observed in sagittal T2-weighted images, HE staining showed that the cavities of the damaged region in the SCI-mod-s group were smaller than those in the SCI-mod-l group at 8 weeks post-SCI (30.54 ± 2.84% and 43.08 ± 2.97%, respectively; *P* < 0.05) (Figures 7A,B). Similar results were obtained with regard to myelin staining and IF staining; compared with the SCI-mod-l group, the SCI-mod-s group showed more area of spared myelin (59.40 ± 2.27% and 51.12 ± 1.718%, respectively; *P* < 0.05) and less area of glial scar (Figures 7C–E). Next, we sought to determine whether the differences of electrophysiology and urinary bladder function exist between the two groups; volatility, latent period, and bladder wall thickness were measured to evaluate the functional recovery of motor conduction and urinary bladder. Compared with the SCI-mod-l group, the SCI-mod-s group showed higher volatility (34.42 ± 8.35 and 17.19 ± 5.58 μV, respectively; *P* < 0.01) and thicker bladder wall (548.70 ± 60.50 and 750.68 ± 186.892 μm, respectively; *P* < 0.05), but no statistical difference of the latent period was observed (6.77 ± 0.68 and 7.81 ± 1.21 ms, respectively; *P* > 0.05) (Figures 7F–J). Together, these data indicated that MRI-based anatomical assessment is consistent with the results of histological analysis and electrophysiological detection.

## Discussion

In order to understand the complex pathological basis of SCI and to develop treatments for improving the quality of patients’ life after SCI, numbers of animal models have been applied in translational studies (Cizkova et al., 2020; Fouad et al., 2020). Most spinal injuries occur from a ventral point in patients, with over 60% located at cervical levels, often caused by indirect traumas resulting in compression injuries (Reier et al., 2012; Musselman et al., 2018). However, due to the advantages of operation, animal care, and ethical issues, dorsal thoracic injury models have been used more widely in preclinical research (Sharif-Alhoseini et al., 2017). Compared with compression injuries and transection injuries, contusion injuries can be graded and produce the cavities that are similar to those observed in a clinical setting (Bresnahan et al., 1991; Basso et al., 1996a). Given the genetic similarities, ethical concerns, and care costs, rodents seem to be the most suitable species for SCI experiments (Sharif-Alhoseini et al., 2017). Among common rat inbred strains, Wistar rats recovered moderately after SCI and could be used as a suitable animal model to observe the difference between the intervention group and control group in functional recovery. Easier urinating procedure leads to a lower occurrence of urinary tract infections and more rapid recovery of bladder function in female rats. These were the reasons why female rats have been used more frequently in SCI studies (Mills et al., 2001; Sedý et al., 2008). In conclusion, as one of the best options for preliminary SCI experiments, the thoracic contusion female Wistar rat model has been applied in this research.

The most suitable tool to estimate structural changes of the spinal cord in clinical study, not possible in the histological approach, is the quantification of changes in non-invasive T2-weighted MRI scans (Huber et al., 2017; Martínez-Pérez et al., 2017; Farhadi et al., 2018; Freund et al., 2019). To facilitate meaningful translation, it is necessary to use MRI as an important method in the experimental design in a preclinical study (Guo et al., 2021). The intramedullary lesion size, measured on sagittal T2-weighted scans, might be a good clinical predictor of recovery, but further studies are required to prove the reliability of that in a rat model (Aarabi et al., 2017). Although the importance of T2-weighted signal changes after injury and its crucial relationship to the neurological outcomes in clinical recovery are well-known, its exact correlation with behavioral outcomes in rat model is not known.

The present study relates MRI-based anatomical evaluation to functional outcome in rats with SCI. To assess a range of injury and adequately cover ceiling effect and floor effect, rats underwent contusion of three different severities followed by MRI, behavioral testing, histological evaluations, and electrophysiological detection. The most commonly used lesion parameters in MRI include LA, LL, LW, and SI. Compared to other parameters in this study, the value of LA might be the best indicators to reflect the severity of injury. Current behavioral testing predominantly relies on the BBB open-field locomotor scale, but a single test scheme is unlikely to be sufficient to evaluate functional recovery after SCI because each testing method has its particular advantages and disadvantages. The deficits not apparent during open-field tests can be revealed by relatively complex tasks or movements of unloaded limbs (Basso et al., 1996b; Metz et al., 2000; Smith et al., 2006b; Sedý et al., 2008). So in this study, the functional outcome was evaluated experimentally by a number of behavioral tests, with BBB score, grid walking, and LSS as the experience-dependent behavioral testing and catwalk gait analysis as the objective behavioral testing. All of them showed strong correlations with the LA measured on sagittal T2-weighted scan behavior at 8 weeks after SCI. In agreement with prior investigations, two rats showed exaggerated changes in MRI but performed similar behavior to others in the same group (Goldstein et al., 1998; Curt, 2012; Dreizin et al., 2015). But this is not enough to mean that the “neuroanatomical–functional” paradox also exists in the thoracic contusion rat model. In addition, LA also can be used as a sensitive indicator to detect subtle differences at a moderate injury level; the results of most behavioral testing, histological evaluations, and electrophysiological detection bear this out. Structural changes of the spinal cord observed in MRI do not correspond to the max contact area and regularity index of the catwalk gait analysis system in moderate injury, which indicated that much better parameters may exist to evaluate the subtle differences of behavioral outcome. Correlation analysis was initially used in this part of the study, but positive results were not obtained possibly because the range of LA and the value of behavioral recovery were narrow. After the permutation test, continuous variables of LA were used to dichotomize the SCI-moderate group into two subgroups by the median. Although the results of group comparison have significant differences, the result of dichotomy has many objective limitations, including massive loss of information, and increases the risk of a positive result being a false positive.

Although radiological and histological assessments presented different results, these two methods have the same efficacy of evaluating the lesion size. Previous studies have shown that lesion size from histological evaluations is well-correlated with outcomes at chronic-phase SCI in rats (Schucht et al., 2002; Hurd et al., 2013). Several studies have also confirmed that lesion size evaluated by T2WI correlated with behavior in SCI rats. However, only one method was applied to evaluate behavior outcomes in these studies (Chitturi et al., 2020; Wilkins et al., 2020; Wu et al., 2020). Taken together, this is the first study to have applied sagittal T2WI parameters along with the combination of multiple different behavioral and histological assessments for exploring the association between spinal morphometry and behavior in a thoracic contusion rat model. The experimental design of three grades of injury severity also can strip out ceiling effects and floor effects. While the advances in SCI imaging techniques are rapid, conventional T2WI parameters are much more accessible to preclinical and clinical research applications, relative to the quantitative MRI technique (Cadotte et al., 2018; Novikov et al., 2018; Vallotton et al., 2019; O’Dell et al., 2020). One of the limitations of this study was the lower sample numbers; besides that, the correlation analysis between MRI-based anatomical assessment and behavioral outcome was only performed on the chronic phase.

## Data Availability Statement

The raw data supporting the conclusions of this article will be made available by the authors, without undue reservation.

## Ethics Statement

The animal study was reviewed and approved by the Ethics Committee of the Institute of Radiation Medicine, Chinese Academy of Medical Sciences & Peking Union Medical College.

## Author Contributions

CX conceived and designed the study and wrote the first draft of the manuscript. ZJ and HQ performed the experiments. SL, HZ, MZ, and SZ performed animal care. ZJ and WJ performed formal analysis of the data. GN and SF were responsible for the integrity of the work as a whole. All authors edited and approved the submitted version of the manuscript.

## Conflict of Interest

The authors declare that the research was conducted in the absence of any commercial or financial relationships that could be construed as a potential conflict of interest.

## Publisher’s Note

All claims expressed in this article are solely those of the authors and do not necessarily represent those of their affiliated organizations, or those of the publisher, the editors and the reviewers. Any product that may be evaluated in this article, or claim that may be made by its manufacturer, is not guaranteed or endorsed by the publisher.

## References

[B1] Aarabi B. Sansur C. Ibrahimi D. Simard J. Hersh D. Le E. (2017). Intramedullary lesion length on postoperative magnetic resonance imaging is a strong predictor of ASIA impairment scale grade conversion following decompressive surgery in cervical spinal cord injury. *Neurosurgery* 80 610–620. 10.1093/neuros/nyw053 28362913 PMC5748932

[B2] Ahuja C. Wilson J. Nori S. Kotter M. Druschel C. Curt A. (2017). Traumatic spinal cord injury. *Nat. Rev. Dis. primers* 3:17018. 10.1038/nrdp.2017.18 28447605

[B3] Barnabé-Heider F. Göritz C. Sabelström H. Takebayashi H. Pfrieger F. Meletis K. (2010). Origin of new glial cells in intact and injured adult spinal cord. *Cell stem cell* 7 470–482. 10.1016/j.stem.2010.07.014 20887953

[B4] Basso D. Beattie M. Bresnahan J. (1995). A sensitive and reliable locomotor rating scale for open field testing in rats. *J. Neurotrauma* 12 1–21. 10.1089/neu.1995.12.1 7783230

[B5] Basso D. Beattie M. Bresnahan J. (1996a). Graded histological and locomotor outcomes after spinal cord contusion using the NYU weight-drop device versus transection. *Exp. Neurol.* 139 244–256. 10.1006/exnr.1996.0098 8654527

[B6] Basso D. Beattie M. Bresnahan J. Anderson D. Faden A. Gruner J. (1996b). MASCIS evaluation of open field locomotor scores: effects of experience and teamwork on reliability. Multicenter animal spinal cord injury study. *J. Neurotrauma* 13 343–359. 10.1089/neu.1996.13.343 8863191

[B7] Behrmann D. Bresnahan J. Beattie M. Shah B. (1992). Spinal cord injury produced by consistent mechanical displacement of the cord in rats: behavioral and histologic analysis. *J. Neurotrauma* 9 197–217. 10.1089/neu.1992.9.197 1474608

[B8] Bresnahan J. Beattie M. Stokes B. Conway K. (1991). Three-dimensional computer-assisted analysis of graded contusion lesions in the spinal cord of the rat. *J. Neurotrauma* 8 91–101. 10.1089/neu.1991.8.91 1870139

[B9] Cadotte D. Akbar M. Fehlings M. Stroman P. Cohen-Adad J. (2018). What has been learned from magnetic resonance imaging examination of the injured human spinal cord: a canadian perspective. *J. Neurotrauma* 35 1942–1957. 10.1089/neu.2018.5903 30074873

[B10] Chitturi J. Sanganahalli B. Herman P. Hyder F. Ni L. Elkabes S. (2020). Association between magnetic resonance imaging-based spinal morphometry and sensorimotor behavior in a hemicontusion model of incomplete cervical spinal cord injury in rats. *Brain Connect.* 10 479–489. 10.1089/brain.2020.0812 32981350 PMC7698856

[B11] Cizkova D. Murgoci A. Cubinkova V. Humenik F. Mojzisova Z. Maloveska M. (2020). Spinal cord injury: animal models, imaging tools and the treatment strategies. *Neurochem. Res.* 45 134–143. 10.1007/s11064-019-02800-w 31006093

[B12] Curt A. (2012). The translational dialogue in spinal cord injury research. *Spinal Cord* 50 352–357. 10.1038/sc.2011.113 22064661

[B13] Dalkilic T. Fallah N. Noonan V. Salimi Elizei S. Dong K. Belanger L. (2018). Predicting injury severity and neurological recovery after acute cervical spinal cord injury: a comparison of cerebrospinal fluid and magnetic resonance imaging biomarkers. *J. Neurotrauma* 35 435–445. 10.1089/neu.2017.5357 29037121

[B14] Dreizin D. Kim W. Kim J. Boscak A. Bodanapally U. Munera F. (2015). Will the real SCIWORA please stand up? Exploring clinicoradiologic mismatch in closed spinal cord injuries. *AJR. Am. J. Roentgenol.* 205 853–860. 10.2214/AJR.14.13374 26397336

[B15] Fan B. Wei Z. Yao X. Shi G. Cheng X. Zhou X. (2018). Microenvironment imbalance of spinal cord injury. *Cell Transplant.* 27 853–866. 10.1177/0963689718755778 29871522 PMC6050904

[B16] Farhadi H. Kukreja S. Minnema A. Vatti L. Gopinath M. Prevedello L. (2018). Impact of admission imaging findings on neurological outcomes in acute cervical traumatic spinal cord injury. *J. Neurotrauma* 35 1398–1406. 10.1089/neu.2017.5510 29361876 PMC13175224

[B17] Fawcett J. Curt A. Steeves J. Coleman W. Tuszynski M. Lammertse D. (2007). Guidelines for the conduct of clinical trials for spinal cord injury as developed by the ICCP panel: spontaneous recovery after spinal cord injury and statistical power needed for therapeutic clinical trials. *Spinal Cord* 45 190–205. 10.1038/sj.sc.3102007 17179973

[B18] Fouad K. Ng C. Basso D. (2020). Behavioral testing in animal models of spinal cord injury. *Exp. Neurol.* 333:113410. 10.1016/j.expneurol.2020.113410 32735871 PMC8325780

[B19] Fouad K. Popovich P. G. Kopp M. A. Schwab J. M. (2021). The neuroanatomical-functional paradox in spinal cord injury. *Nat. Rev. Neurol.* 17 53–62. 10.1038/s41582-020-00436-x 33311711 PMC9012488

[B20] Freund P. Seif M. Weiskopf N. Friston K. Fehlings M. Thompson A. (2019). MRI in traumatic spinal cord injury: from clinical assessment to neuroimaging biomarkers. *Lancet Neurol.* 18 1123–1135. 10.1016/S1474-4422(19)30138-3 31405713

[B21] Goldstein B. Hammond M. Stiens S. Little J. (1998). Posttraumatic syringomyelia: profound neuronal loss, yet preserved function. *Arch. Phys. Med. Rehabil.* 79 107–112. 10.1016/s0003-9993(98)90217-9 9440427

[B22] Göritz C. Dias D. Tomilin N. Barbacid M. Shupliakov O. Frisén J. (2011). A pericyte origin of spinal cord scar tissue. *Science* 333 238–242. 10.1126/science.1203165 21737741

[B23] Guo X. Feng Y. Sun T. Feng S. Tang J. Chen L. (2021). Clinical guidelines for neurorestorative therapies in spinal cord injury (2021 China version). *J. Neurorestoratology* 9 31–49. 10.26599/jnr.2021.9040003

[B24] Hamers F. Koopmans G. Joosten E. (2006). CatWalk-assisted gait analysis in the assessment of spinal cord injury. *Journal of neurotrauma* 23 537–548. 10.1089/neu.2006.23.537 16629635

[B25] Huang H. Young W. Skaper S. Chen L. Moviglia G. Saberi H. (2020). Clinical neurorestorative therapeutic guidelines for spinal cord injury (IANR/CANR version 2019). *J. Orthop. Translat.* 20 14–24. 10.1016/j.jot.2019.10.006 31908929 PMC6939117

[B26] Huber E. Lachappelle P. Sutter R. Curt A. Freund P. (2017). Are midsagittal tissue bridges predictive of outcome after cervical spinal cord injury? *Ann. Neurol.* 81 740–748. 10.1002/ana.24932 28393423

[B27] Hurd C. Weishaupt N. Fouad K. (2013). Anatomical correlates of recovery in single pellet reaching in spinal cord injured rats. *Exp. Neurol.* 247 605–614. 10.1016/j.expneurol.2013.02.013 23470552

[B28] James S. L. Theadom A. Ellenbogen R. G. Bannick M. S. Montjoy-Venning W. Lucchesi L. R. (2019). Global, regional, and national burden of traumatic brain injury and spinal cord injury, 1990–2016: a systematic analysis for the global burden of disease study 2016. *Lancet Neurol.* 18 56–87. 10.1016/S1474-4422(18)30415-0 30497965 PMC6291456

[B29] Kirshblum S. Snider B. Rupp R. Read M. (2020). Updates of the international standards for neurologic classification of spinal cord injury: 2015 and 2019. *Phys. Med. Rehabil. Clin. N. Am.* 31 319–330. 10.1016/j.pmr.2020.03.005 32624097

[B30] Kirshblum S. Waring W. Biering-Sorensen F. Burns S. Johansen M. Schmidt-Read M. (2011). Reference for the 2011 revision of the international standards for neurological classification of spinal cord injury. *J. Spinal Cord Med.* 34 547–554. 10.1179/107902611X13186000420242 22330109 PMC3232637

[B31] Le E. Aarabi B. Hersh D. Shanmuganathan K. Diaz C. Massetti J. (2015). Predictors of intramedullary lesion expansion rate on MR images of patients with subaxial spinal cord injury. *J. Neurosurg. Spine* 22 611–621. 10.3171/2014.10.SPINE14576 25746115

[B32] Marino R. Ditunno J. Donovan W. Maynard F. (1999). Neurologic recovery after traumatic spinal cord injury: data from the model spinal cord injury systems. *Arch. Phys. Med. Rehabil.* 80 1391–1396. 10.1016/s0003-9993(99)90249-6 10569432

[B33] Martínez-Pérez R. Cepeda S. Paredes I. Alen J. Lagares A. M. R. I. (2017). Prognostication factors in the setting of cervical spinal cord injury secondary to trauma. *World Neurosurg.* 101 623–632. 10.1016/j.wneu.2017.02.034 28216400

[B34] Metz G. Merkler D. Dietz V. Schwab M. Fouad K. (2000). Efficient testing of motor function in spinal cord injured rats. *Brain Res.* 883 165–177. 10.1016/s0006-8993(00)02778-5 11074045

[B35] Mills C. Hains B. Johnson K. Hulsebosch C. (2001). Strain and model differences in behavioral outcomes after spinal cord injury in rat. *J. Neurotrauma* 18 743–756. 10.1089/089771501316919111 11526981

[B36] Musselman K. Shah M. Zariffa J. (2018). Rehabilitation technologies and interventions for individuals with spinal cord injury: translational potential of current trends. *J. Neuroeng. Rehabil.* 15:40. 10.1186/s12984-018-0386-7 29769082 PMC5956557

[B37] Norenberg M. Smith J. Marcillo A. (2004). The pathology of human spinal cord injury: defining the problems. *J. Neurotrauma* 21 429–440. 10.1089/089771504323004575 15115592

[B38] Novikov D. Kiselev V. Jespersen S. (2018). On modeling. *Magn. Reson. Med.* 79 3172–3193. 29493816 10.1002/mrm.27101 PMC5905348

[B39] O’Dell D. Weber K. Berliner J. Elliott J. Connor J. Cummins D. (2020). Midsagittal tissue bridges are associated with walking ability in incomplete spinal cord injury: a magnetic resonance imaging case series. *J. Spinal Cord Med.* 43 268–271. 10.1080/10790268.2018.1527079 30346248 PMC7054908

[B40] Peng Z. Li X. Fu M. Zhu K. Long L. Zhao X. (2019). Inhibition of Notch1 signaling promotes neuronal differentiation and improves functional recovery in spinal cord injury through suppressing the activation of Ras homolog family member A. *J. Neurochem.* 150 709–722. 10.1111/jnc.14833 31339573

[B41] Reier P. Lane M. Hall E. Teng Y. Howland D. (2012). Translational spinal cord injury research: preclinical guidelines and challenges. *Handb. Clin. Neurol.* 109 411–433. 10.1016/B978-0-444-52137-8.00026-7 23098728 PMC4288927

[B42] Robac A. Neveu P. Hugede A. Garrido E. Nicol L. Delarue Q. (2021). Repetitive trans spinal magnetic stimulation improves functional recovery and tissue repair in contusive and penetrating spinal cord injury models in rats. *Biomedicines* 9:1827. 10.3390/biomedicines9121827 34944643 PMC8698720

[B43] Rowland J. Hawryluk G. Kwon B. Fehlings M. (2008). Current status of acute spinal cord injury pathophysiology and emerging therapies: promise on the horizon. *Neurosurg. Focus* 25:E2. 10.3171/FOC.2008.25.11.E2 18980476

[B44] Schucht P. Raineteau O. Schwab M. Fouad K. (2002). Anatomical correlates of locomotor recovery following dorsal and ventral lesions of the rat spinal cord. *Exp. Neurol.* 176 143–153. 10.1006/exnr.2002.7909 12093091

[B45] Sedý J. Urdzíková L. Jendelová P. Syková E. (2008). Methods for behavioral testing of spinal cord injured rats. *Neurosci. Biobehav. Rev.* 32 550–580. 10.1016/j.neubiorev.2007.10.001 18036661

[B46] Seif M. David G. Huber E. Vallotton K. Curt A. Freund P. (2020). Cervical cord neurodegeneration in traumatic and non-traumatic spinal cord injury. *J. Neurotrauma* 37 860–867. 10.1089/neu.2019.6694 31544628 PMC7071087

[B47] Sharif-Alhoseini M. Khormali M. Rezaei M. Safdarian M. Hajighadery A. Khalatbari M. (2017). Animal models of spinal cord injury: a systematic review. *Spinal Cord* 55 714–721. 10.1038/sc.2016.187 28117332

[B48] Smith R. Burke D. Baldini A. Shum-Siu A. Baltzley R. Bunger M. (2006a). The Louisville Swim Scale: a novel assessment of hindlimb function following spinal cord injury in adult rats. *J. Neurotrauma* 23 1654–1670. 10.1089/neu.2006.23.1654 17115911 PMC2833969

[B49] Smith R. Shum-Siu A. Baltzley R. Bunger M. Baldini A. Burke D. (2006b). Effects of swimming on functional recovery after incomplete spinal cord injury in rats. *J. Neurotrauma* 23 908–919. 10.1089/neu.2006.23.908 16774475 PMC2831776

[B50] Vallotton K. Huber E. Sutter R. Curt A. Hupp M. Freund P. (2019). Width and neurophysiologic properties of tissue bridges predict recovery after cervical injury. *Neurology* 92 e2793–e 2802. 31092621 10.1212/WNL.0000000000007642 PMC6598793

[B51] Walker C. Fry C. Wang J. Du X. Zuzzio K. Liu N. (2019). Functional and histological gender comparison of age-matched rats after moderate thoracic contusive spinal cord injury. *J. Neurotrauma* 36 1974–1984. 30489213 10.1089/neu.2018.6233 PMC6599384

[B52] Wilkins N. Skinner N. Motovylyak A. Schmit B. Kurpad S. Budde M. (2020). Evolution of magnetic resonance imaging as predictors and correlates of functional outcome after spinal cord contusion injury in the rat. *J. Neurotrauma* 37 889–898. 10.1089/neu.2019.6731 31830856 PMC7071026

[B53] Wu T. Byun N. Wang F. Mishra A. Janve V. Chen L. (2020). Longitudinal assessment of recovery after spinal cord injury with behavioral measures and diffusion, quantitative magnetization transfer and functional magnetic resonance imaging. *NMR Biomed.* 33:e4216. 10.1002/nbm.4216 31943383 PMC7155919

[B54] Zipser C. Margetis K. Pedro K. Curt A. Fehlings M. Sadler I. (2021). Increasing awareness of degenerative cervical myelopathy: a preventative cause of non-traumatic spinal cord injury. *Spinal Cord* 59 1216–1218. 10.1038/s41393-021-00711-8 34628477 PMC8560634

