# Supplementary material for: Correlation Analysis Between Magnetic Resonance Imaging-Based Anatomical Assessment and Behavioral Outcome in a Rat Contusion Model of Chronic Thoracic Spinal Cord Injury
Source: Front Neurosci. 2022 Apr 21;16:838786. doi: 10.3389/fnins.2022.838786 (PMC9069114; doi:10.3389/fnins.2022.838786)
Supplement: Supplementary file 1 [file Data_Sheet_1.docx]

**
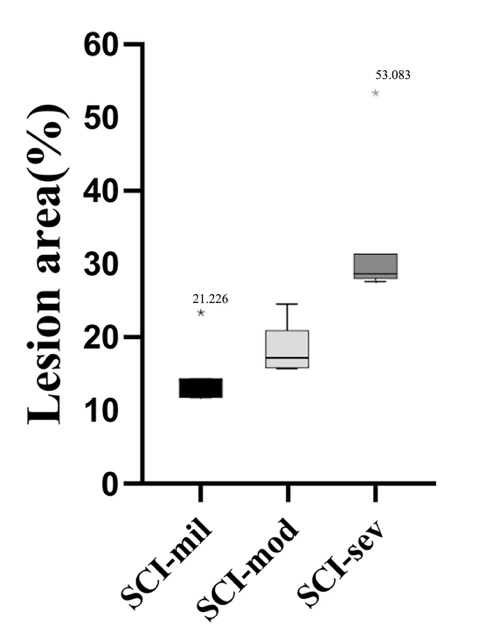
**

**Supplementary Figure 1.** Box plot analysis of lesion area in three groups.

**Supplementary Table 1.** The value of lesion area measured by MRI and the corresponding data of behavior testing in three groups

| **Group** | **Number** | **Lesion area（%）** |  | **Subjective methods of behavioral testing** | | |  | **Catwalk gait analysis system** | | | | | | |
| --- | --- | --- | --- | --- | --- | --- | --- | --- | --- | --- | --- | --- | --- | --- |
|  |  |  |  | **BBB score** | **Grid-walking Test** | **LSS scores** |  | **Swing time (s)** | **Max contact area (cm^2^)** | | **Regularity**  **index (%)** | | **Print position (cm)** | |
| **SCI-mil** | 1 | 0.09 |  | 16 | 0.27 | 12 |  | 0.23 | | 0.87 | | 87.07 | | 3.21 |
|  | 2 | 0.13 |  | 15 | 0.25 | 11 |  | 0.10 | | 0.98 | | 87.67 | | 4.74 |
|  | 3 | 0.12 |  | 16 | 0.30 | 12 |  | 0.13 | | 1.06 | | 84.64 | | 5.24 |
|  | 4 | 0.21 |  | 16 | 0.12 | 12 |  | 0.10 | | 0.86 | | 93.73 | | 4.18 |
|  | 5 | 0.09 |  | 16 | 0.38 | 11 |  | 0.12 | | 1.04 | | 88.57 | | 4.71 |
| **SCI-mod** | 1 | 0.19 |  | 10 | 0.67 | 7 |  | 0.60 | | 0.42 | | 63.59 | | 9.15 |
|  | 2 | 0.13 |  | 12 | 0.55 | 9 |  | 0.36 | | 0.73 | | 68.69 | | 5.55 |
|  | 3 | 0.22 |  | 10 | 0.63 | 8 |  | 0.71 | | 0.72 | | 74.44 | | 10.15 |
|  | 4 | 0.15 |  | 12 | 0.35 | 9 |  | 0.49 | | 0.59 | | 57.05 | | 7.00 |
|  | 5 | 0.13 |  | 10 | 0.48 | 8 |  | 0.32 | | 0.28 | | 76.36 | | 7.96 |
| **SCI-sev** | 1 | 0.27 |  | 8 | 0.72 | 2 |  | 0.86 | | 0.30 | | 43.29 | | 12.15 |
|  | 2 | 0.53 |  | 8 | 0.84 | 3 |  | 1.19 | | 0.20 | | 0.00 | | 12.38 |
|  | 3 | 0.30 |  | 9 | 0.62 | 4 |  | 1.19 | | 0.36 | | 29.40 | | 10.81 |
|  | 4 | 0.20 |  | 8 | 0.75 | 5 |  | 0.52 | | 0.37 | | 37.12 | | 12.02 |
|  | 5 | 0.26 |  | 8 | 0.70 | 0 |  | 0.64 | | 0.23 | | 22.22 | | 8.13 |

**Supplementary Table 2.** Comparison between two SCI-mod groups on lesion area and behavior testing

| **Group** | **Number** | **Lesion area**  **（%）** |  | **Subjective methods of behavioral testing** | | |  | **Catwalk gait analysis system** | | | |
| --- | --- | --- | --- | --- | --- | --- | --- | --- | --- | --- | --- |
|  |  |  |  | **BBB score** | **Grid-walking** | **LSS scores** |  | **Swing time**  **(s)** | **Max contact area (cm^2^)** | **Regularity index (%)** | **Print position (cm)** |
| **SCI-mod-s** | 1 | 0.1332 |  | 11 | 0.50 | 7 |  | 0.24 | 0.33 | 80.81 | 6.51 |
|  | 2 | 0.1468 |  | 13 | 0.42 | 9 |  | 0.11 | 1.05 | 87.07 | 5.64 |
|  | 3 | 0.1312 |  | 12 | 0.55 | 9 |  | 0.35 | 0.73 | 86.01 | 5.55 |
|  | 4 | 0.1465 |  | 12 | 0.35 | 9 |  | 0.14 | 0.59 | 85.07 | 7.00 |
|  | 5 | 0.1321 |  | 10 | 0.48 | 8 |  | 0.31 | 0.28 | 76.36 | 7.96 |
|  | 6 | 0.1466 |  | 11 | 0.60 | 8 |  | 0.28 | 0.30 | 57.05 | 6.72 |
| **SCI-mod-l** | 1 | 0.1652 |  | 11 | 0.61 | 8 |  | 0.32 | 0.36 | 90.00 | 6.43 |
|  | 2 | 0.1860 |  | 10 | 0.67 | 7 |  | 0.60 | 0.42 | 63.59 | 9.15 |
|  | 3 | 0.2246 |  | 10 | 0.63 | 8 |  | 0.71 | 0.72 | 74.44 | 10.15 |
|  | 4 | 0.1566 |  | 9 | 0.69 | 1 |  | 0.91 | 0.30 | 45.71 | 9.87 |
|  | 5 | 0.1505 |  | 9 | 0.67 | 4 |  | 1.15 | 0.24 | 68.69 | 10.17 |
|  | 6 | 0.1519 |  | 10 | 0.67 | 6 |  | 0.49 | 0.35 | 74.87 | 10.04 |
